# Ultrasonographic Quantification of Reduction in Subcutaneous Fat Thickness Following Large-Volume Liposuction

**DOI:** 10.7759/cureus.103904

**Published:** 2026-02-19

**Authors:** Sanjibani Sudha, Biswanu S Biswal, Vivek Kumar, Lalit Choudhary, Nandita Dimri, Ritesh Panda

**Affiliations:** 1 Burns and Plastic Surgery, Institute of Medical Sciences and Sum Hospital, Bhubaneswar, IND; 2 Urology, Institute of Medical Sciences and Sum Hospital, Bhubaneswar, IND; 3 Plastic and Reconstructive Surgery, Ganga Ram Institute of Postgraduate Medical Education and Research, New Delhi, IND; 4 Radiodiagnosis, Ganga Ram Institute of Postgraduate Medical Education and Research, New Delhi, IND; 5 Trauma and Emergency/Plastic and Reconstructive Surgery, All India Institute of Medical Sciences, Bhubaneswar, IND

**Keywords:** aesthetic surgery, body composition assessment, large-volume liposuction, subcutaneous fat thickness, ultrasonography or ultrasound, waist circumference reduction

## Abstract

Background: Large‑volume liposuction is widely performed for abdominal contouring, yet objective, site‑specific quantification of subcutaneous fat reduction remains limited. Ultrasonography (USG) offers a practical, radiation‑free method for serial assessment, but its postoperative utility is under‑reported.

Objective: This study aimed to quantify changes in abdominal subcutaneous fat thickness following large‑volume liposuction using USG and to evaluate associated changes in waist circumference, complications, and patient satisfaction.

Methods: A prospective observational study was conducted on 50 patients (21-45 years) undergoing large‑volume abdominal liposuction (>5 L aspirate). Subcutaneous fat thickness was measured ultrasonographically at four standardized abdominal sites (supra‑umbilical (S), infra‑umbilical (I), right (R), and left (L)), pre‑operatively, one week, one month, and six months. Waist circumference was recorded at six months. Complications and patient satisfaction were documented. Repeated‑measures ANOVA assessed changes over time, and Pearson correlations evaluated associations between fat‑thickness reduction and waist‑circumference change.

Results: Significant reductions in subcutaneous fat thickness were observed at all sites (p < 0.001), with mean reductions of 36%-44% at six months. Early maximal reductions at one week were followed by partial rebound at one month and stabilization by six months, reflecting edema resolution and tissue remodeling. Waist circumference decreased by a mean of 9.82 cm (≈10%). Correlations between waist‑circumference reduction and ultrasonographic fat‑thickness changes were weak and non‑significant, indicating that circumferential measures do not reliably reflect localized fat removal. Postoperative seromas/hematomas occurred in 84% at one week and resolved by one month. No major complications were observed. Patient satisfaction at six months was 96%.

Conclusion: Large‑volume abdominal liposuction produces substantial and durable reductions in subcutaneous fat thickness and waist circumference. USG is a reliable, accessible, and cost‑effective modality for objective, site‑specific postoperative assessment, outperforming waist circumference in detecting localized fat‑layer changes. Larger studies with extended follow‑up and comparative imaging may further refine its role in body‑contouring evaluation.

## Introduction

Cosmetic surgery uniquely strives to achieve body transformation by addressing not a disease, but a mind's quest for youth and beauty, and liposuction is the medium of artistic expression. Liposuction is a surgical technique that removes excess fat deposits from specific areas of the body using suction cannulas inserted through small incisions in the skin. Large-volume liposuction is defined as the removal of a total aspirate volume of 5 L or more during a single procedure (including fat and infiltrated wetting solution) [1]. Liposuction aims to remove target fat, leaving the desired contour and a smooth transition between suctioned and non-suctioned areas. There are various types of liposuctions, such as suction-assisted liposuction (SAL), ultrasound-assisted liposuction (UAL), power-assisted liposuction (PAL), water-assisted liposuction, and laser-assisted liposuction.

Ultrasonography (USG) can be used in clinical practice as a diagnostic tool, when utilized for imaging, or as a therapeutic modality. Most people are familiar with the biomedical diagnostic application of USG, but the use of USG to measure fat and muscle thickness in humans is less well-known. A review article by Baselga et al. [2] discussed the use of USG in clinical body composition assessment, noting that it can measure both subcutaneous and visceral fat, as well as muscle compartments. Liposuction is a standard method for reducing subcutaneous fat; however, the extent of this reduction has not been rigorously quantified. Despite a nearly 50-year history of USG being used to measure subcutaneous adipose tissue, this technology appears to be used far less frequently than the previously mentioned methods for assessing body composition, and many students, researchers, and clinicians are unfamiliar with its usefulness and versatility as a body composition assessment tool. This study will evaluate the serial changes and reduction in the thickness of the subcutaneous fat layer of the abdomen using ultrasonography.

## Materials and methods

The present study was a prospective observational study aimed at assessing preoperative and postoperative changes in subcutaneous fat following liposuction, as well as the precise reduction in abdominal subcutaneous fat thickness.

The study was conducted over three and a half years, from August 1, 2016, to January 31, 2020, after obtaining approval from the Sir Ganga Ram Hospital Ethics Committee (EC/09/14/731). Fifty patients admitted to the Department of Plastic and Reconstructive Surgery at a multispecialty hospital in New Delhi, India, who presented with varying degrees of abdominal lipodystrophy were included. Established methodologies for health research and previous clinical studies in body contouring guided the sample size determination. The reliability of USG in quantifying subcutaneous fat reduction after liposuction has been validated in earlier studies. The study population consisted of patients aged 21-45 years with truncal obesity and no other significant illnesses. Only those meeting the predefined inclusion and exclusion criteria were eligible to participate.

Inclusion criteria

Patients with localized fat deposits resistant to diet and exercise, patients classified as ASA I or II according to the American Society of Anesthesiologists Physical Status Classification System, and patients with realistic expectations regarding the outcome of the procedure were included in the study.

Exclusion criteria

Exclusion criteria included patients undergoing concomitant procedures; those with significant medical diseases such as diabetes, cardiac, renal, hepatic, gastrointestinal, and endocrine diseases; individuals uncontrolled on medications (ASA III and IV); patients who inherited or acquired coagulopathies, including those on anticoagulants; patients with neurologic and psychiatric disorders; and pregnant or breastfeeding women.

Outcome of the study and sample size

The primary outcome was the reduction in subcutaneous fat thickness at four abdominal sites (supra-umbilical (S), infra-umbilical (I), right lateral (R), and left lateral (L)), measured by USG. Secondary outcomes included reduction in waist circumference (WC), patient satisfaction, and postoperative complications. WC was measured using a non-stretchable measuring tape at the midpoint between the lower margin of the last palpable rib and the top of the iliac crest. Measurements were taken with the patient standing erect, feet shoulder-width apart, arms relaxed, and at the end of normal expiration. Measurements were recorded at the following time points: preoperative, one week, one month, and six months postoperative. The sample size calculation targeted the paired difference. Key inputs for the paired t-test formula included: α (two-sided) = 0.05; desired power (1−β) = 0.80 (80%) for planning (observed (post hoc) power was also reported for the recruited sample); and expected mean paired difference (Δ), defined as the minimal clinically important difference. Although prior ultrasound studies of non-surgical localized fat reduction reported reductions in the range of a few millimeters, liposuction produces substantially larger reductions. Therefore, a conservative and clinically meaningful difference of 10 mm was used for planning. The estimated standard deviation of paired differences was set at 15 mm. While published ultrasound studies report single-digit standard deviations for non-surgical interventions and somewhat higher variability for surgical procedures, a value of 15 mm was considered conservative for liposuction studies. Written informed consent was obtained from all participants prior to study commencement, and strict confidentiality was maintained throughout.

**Figure 1:**
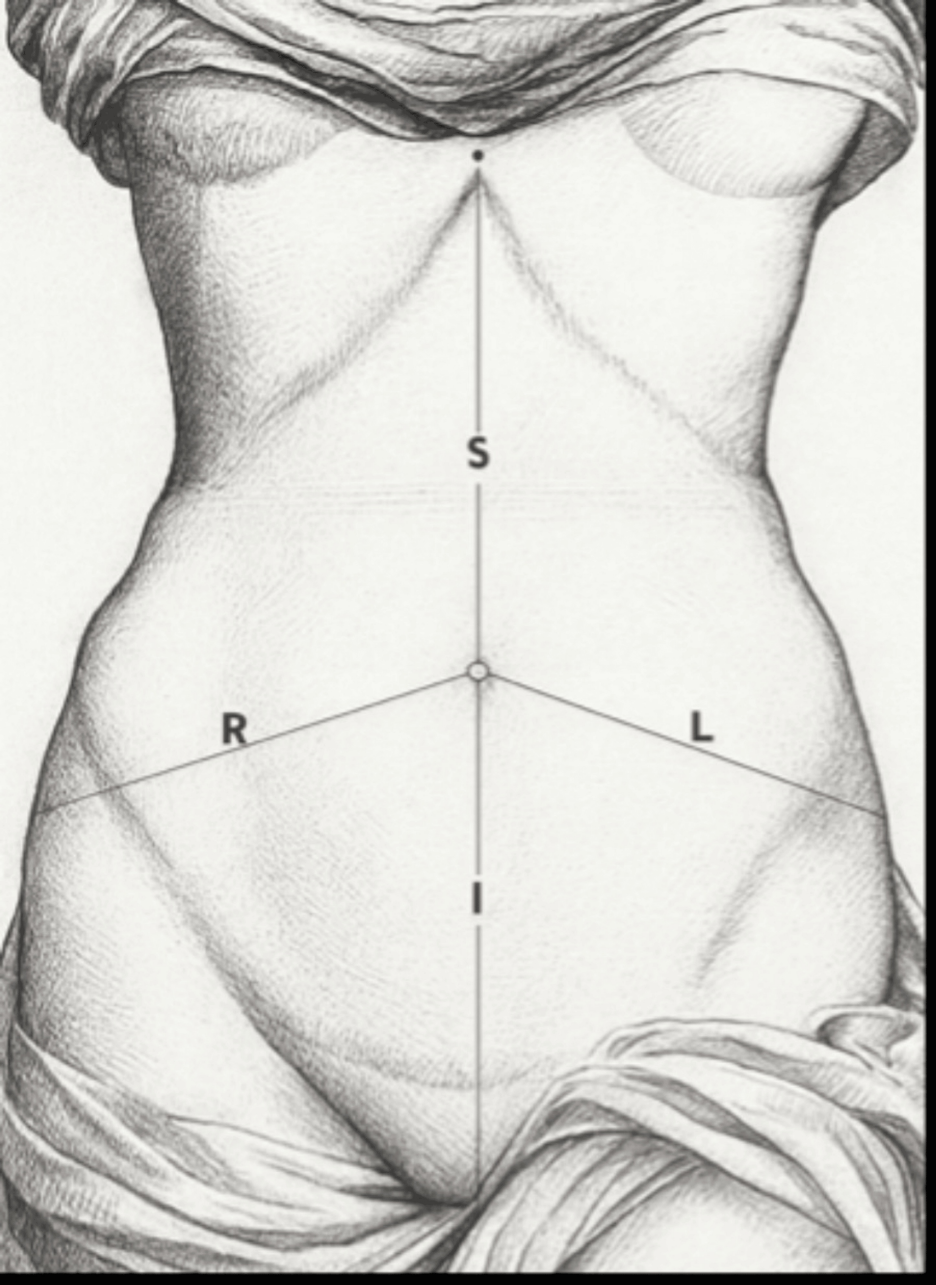
Markings of the points for ultrasonographic measurements S: midpoint of the xiphisternum and umbilicus. I: midpoint of the umbilicus and pubic symphysis. R (right), L (left): midpoint of the anterior superior iliac spine (ASIS) and umbilicus. Image Credit: Authors' original creation.

**Figure 2:**
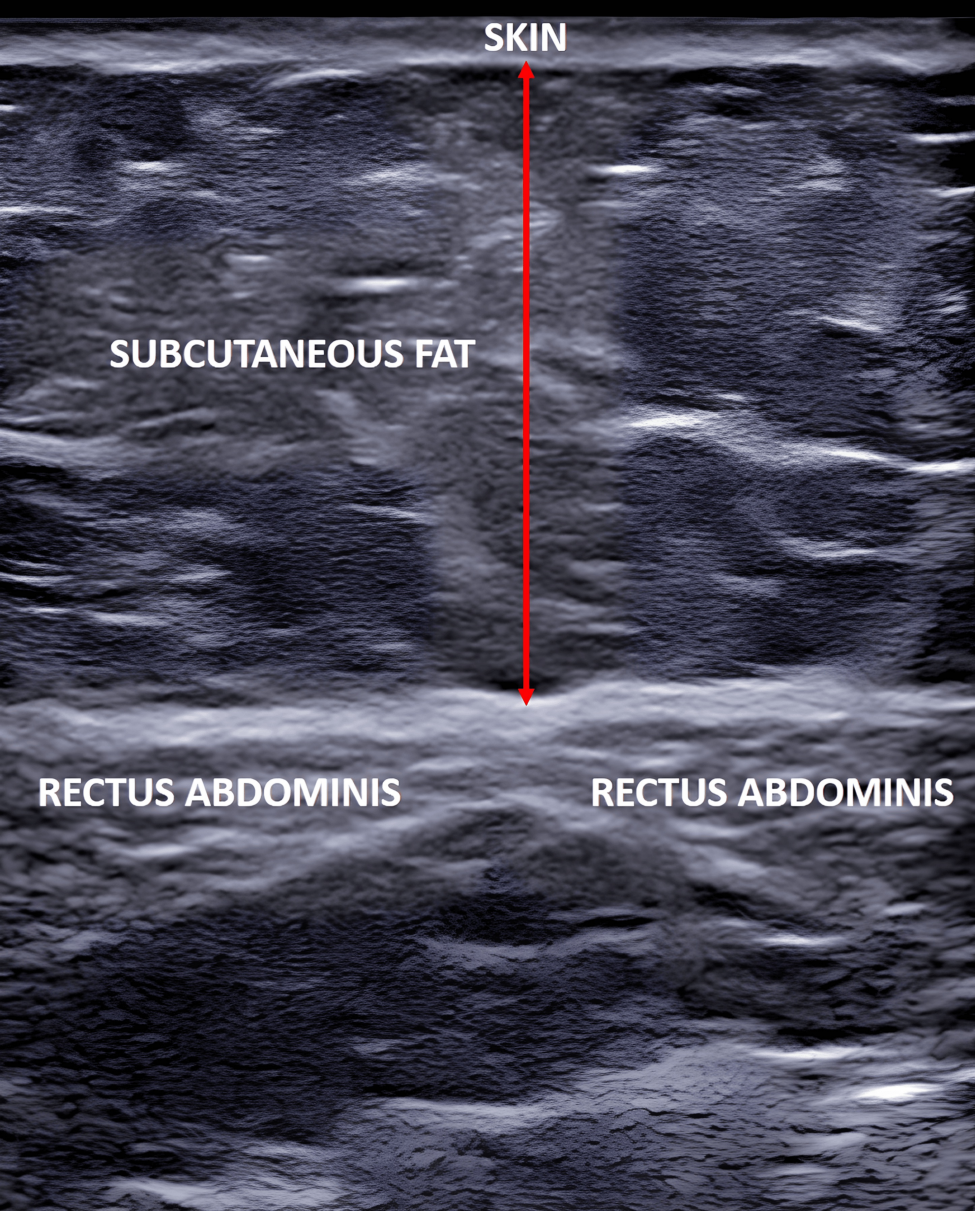
Ultrasonographic image of the subcutaneous fat of the abdomen

Skin markings for ultrasonographic assessment were defined as follows: midpoint between the xiphisternum and umbilicus (S), midpoint between the umbilicus and pubic symphysis (I), and midpoint between the anterior superior iliac spine (ASIS) and the umbilicus on both the right (R) and left (L) sides (Figure 1). A thorough preoperative systemic examination was performed by a single operator. Ultrasonographic measurements were obtained using a high-frequency linear probe (7-12 MHz), which provides optimal resolution for superficial soft-tissue assessment (Figure 2). All scans were performed with the patient in the supine position without abdominal muscle contraction. To minimize operator-dependent variability, all examinations were conducted by the same radiologist with more than five years of experience in musculoskeletal USG. Consistent probe pressure was maintained by applying only the weight of the transducer, avoiding compression that could artificially reduce fat thickness measurements. To assess reproducibility, a subset of 10 randomly selected patients underwent repeat measurements by a second radiologist. Inter-observer variability was minimal (<5% variation), supporting the reliability of the measurement protocol.

Power-assisted liposuction was performed under general anesthesia with the patient in the supine position after infiltration of a tumescent solution containing 50 mL of 1% lidocaine, 1 mL of adrenaline (1:1000), and 12.5 mL of 8.4% sodium bicarbonate, diluted in 1 L of 0.9% normal saline. Early ambulation was initiated on postoperative day (POD) 1. A pressure garment was applied from PODs 3-4 onward and continued for 6-8 weeks. Postoperative massage was initiated during the third postoperative week. USG for assessment of subcutaneous changes was repeated at one week, one month, and six months postoperatively. Postoperative ultrasonography at one week and one month was systematically evaluated for fluid collections using standardized criteria, defined as hypoechoic areas > 1 cm located between the subcutaneous fat and fascia. Reduction in WC was assessed at six months postoperatively.

Statistical analysis

The results were recorded in Microsoft Excel (Microsoft Corp., Redmond, WA, USA). Statistical analysis was performed using SPSS, Version 17.0 (SPSS Inc., Chicago, IL, USA). Continuous variables are presented as mean ± standard deviation (SD), and categorical variables are summarized as frequencies and percentages. Within-subject comparisons over time were analyzed using repeated-measures ANOVA, followed by Bonferroni post hoc tests (pairwise comparisons based on paired t-test logic, including preoperative vs. 6-month comparisons). Assumptions were verified prior to analysis: normality was assessed using Q-Q plots and the Shapiro-Wilk test, and sphericity was evaluated using Mauchly’s test, with Greenhouse-Geisser correction applied when the assumption was violated. Effect sizes were reported as partial eta squared (η²) for ANOVA and Cohen’s d for paired comparisons. Correlations between variables were assessed using Pearson’s correlation coefficient, or Spearman’s rank correlation when normality assumptions were not met. Correlation analysis was performed to evaluate the relationship between percentage changes in WC and ultrasonographic measurements at sites S, I, R, and L. The degree of reduction in WC and body weight was also evaluated.

## Results

A total of 50 patients were evaluated. The most common age group undergoing abdominal liposuction was 26-30 years, comprising 19 (38%) patients, while the least common age group was 21-25 years, with 6 (12%) patients. Females outnumbered males, with 35 (70%) women and 15 (30%) men. This demographic distribution suggests that abdominal liposuction is particularly sought by younger adults, especially women, possibly reflecting aesthetic preferences and social trends in body contouring. Participants represented diverse occupational backgrounds, including business professionals, homemakers, and students (Table 1). The mean total aspirate volume was 7,858 mL, with a median of 7,250 mL and a standard deviation of 2,190 mL. The mean fat component was estimated at 65%-75% of the total aspirate, based on standard tumescent ratios (approximately 5,100-5,900 mL of pure fat), consistent with the definition of large-volume liposuction. The wide range and standard deviation reflect variability in patient presentation and the extent of truncal adiposity treated.

**Table 1:** Baseline parameters of the study participants (N = 50)

Parameters	n, %
Age distribution (years)	
21-25	6 (12%)
26-30	19 (38%)
31-35	8 (16%)
36-40	10 (20%)
>40	7 (14%)
Gender distribution	
Female	35 (70%)
Male	15 (30%)
Occupation	
Businessman	3 (6%)
Working	12 (24%)
Housewife	8 (16%)
Teacher	5 (10%)
Software engineer	7 (14%)
Engineer	5 (10%)
Student	10 (20%)

**Figure 3:**
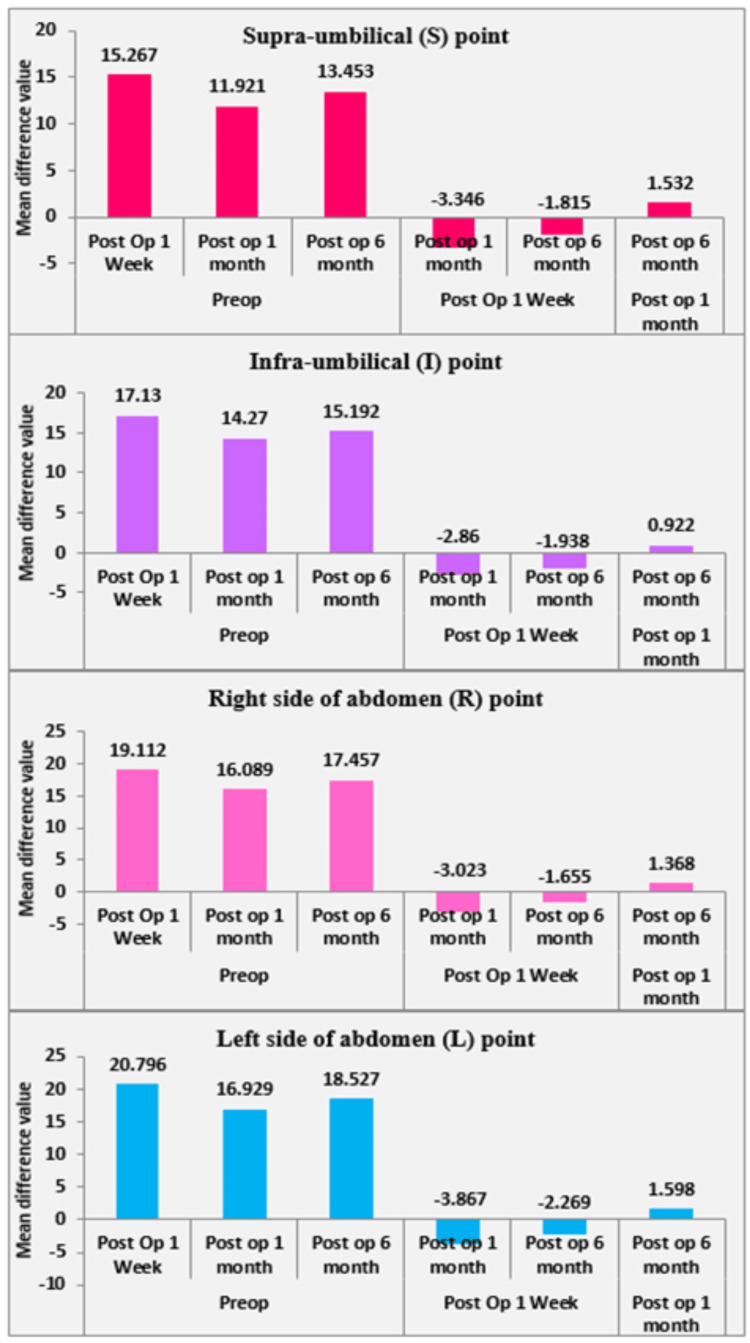
Trends in the mean reduction of subcutaneous fat thickness at various points

Table 2 presents the mean residual subcutaneous fat thickness (in millimeters) measured at four abdominal sites (S, I, R, and L), assessed preoperatively and at subsequent follow-up visits. WC was measured at six months postoperatively. At six months, the greatest percentage reduction in abdominal subcutaneous fat thickness was observed at the L site (44.14%), followed by the R site (42.88%), the I site (39.9%), and the least reduction at the S site (36.08%). Figure 3 illustrates the trends in mean reduction in subcutaneous fat thickness across the four sites. At the S site, the mean reduction was 15.26 mm at one week postoperatively, 11.92 mm at one month, and 13.45 mm at six months. At the I site, the mean reduction was 17.13 mm at one week, 14.27 mm at one month, and 15.19 mm at six months following large-volume liposuction. At the R site, the mean reduction was 19.11 mm at one week, 16.08 mm at one month, and 17.45 mm at six months. Similarly, at the L site, the mean reduction was 20.79 mm at one week, 16.92 mm at one month, and 18.52 mm at six months. The mean reduction in WC at the level of the umbilicus was 9.82 cm, corresponding to 9.7% of the preoperative WC (Table 2). Correlation analysis was performed to determine whether reductions in WC were associated with proportional reductions in subcutaneous fat thickness at the four abdominal sites. As shown in Table 3, all correlations were weak and statistically non-significant. The S site demonstrated a very weak positive correlation (r = 0.107, p = 0.459), whereas the I site showed a very weak negative correlation (r = −0.089, p = 0.540). The R and L sites exhibited negligible correlations (r = 0.045 and r = 0.044, respectively; p > 0.75). These findings indicate that changes in WC did not reliably correspond to localized reductions in subcutaneous fat thickness, underscoring the greater sensitivity of ultrasonography for site-specific assessment. At six months post-liposuction, the subcutaneous fat demonstrated an appearance similar to the preoperative state, characterized as a well-organized, hypoechoic structure with intervening septae. The dermis and underlying fascia also appeared comparable to the preoperative condition.

**Table 2:** Correlation between percentage reduction in WC and percentage reductions in S, I, R, and L sites Note: The table reports correlation coefficients and p‑values derived from percentage‑reduction data; raw percentage values are not shown. WC: waist circumference, S: supra-umbilical, I: infra-umbilical, R: right abdominal, L: left abdominal.

		Percentage reduction in S	Percentage reduction in I	Percentage reduction in R	Percentage reduction in L
Percentage reduction in WC	Correlation coefficient	0.107	-0.089	0.045	0.044
	p-value	0.459	0.54	0.757	0.761
	N	50	50	50	50

**Table 3:** Mean residual subcutaneous fat thickness (mm) and waist circumference (cm) measurements at various points (N = 50) WC: waist circumference, S: supra-umbilical, I: infra-umbilical, R: right abdominal, L: left abdominal.

Subcutaneous fat thickness	Pre-op	1-week post-op	1-month post-op	6-month post-op
S	37.27	22.01	25.35	23.82
I	38.09	20.96	23.82	22.89
R	40.71	21.6	24.62	23.25
L	41.98	21.18	25.05	23.45
WC	101.4	-	-	91.58

Postoperative seromas/hematomas occurred in 42 of 50 patients (84%) and were detected on routine ultrasonography at one week as hypoechoic fluid collections between the subcutaneous fat and fascia. All cases resolved either spontaneously or with serial aspiration by one month, resulting in a 100% resolution rate among the 42 affected patients. No cases of skin necrosis, major contour irregularities, thromboembolic events, or systemic complications were observed. High patient satisfaction rates and minimal complications suggest that the procedure is effective and safe when performed under appropriate conditions. At six months postoperatively, the abdominal subcutaneous fat layer appeared as well-organized as in the preoperative period. Subcutaneous fat thickness reductions were greatest at one week postoperatively (S: 15.26 mm; I: 17.13 mm; R: 19.11 mm; L: 20.79 mm), followed by partial rebound at one month and stabilization at six months (S: 13.45 mm; I: 15.19 mm; R: 17.45 mm; L: 18.52 mm). This pattern suggests that early postoperative changes were influenced by edema and fluid shifts, whereas longer-term outcomes reflect actual fat removal and tissue remodeling. The persistence of reduced fat thickness at six months supports the durability of liposuction results.

At six months, 48 patients (96%) reported satisfaction with the procedure. The observed fat reduction and improvement in WC are consistent with published outcomes for large-volume liposuction. The use of ultrasonography for measurement is supported by recent literature highlighting its reliability and practicality compared with other imaging modalities, such as CT or MRI. The pattern of fat reduction across abdominal sites and the high satisfaction rates are also consistent with findings from systematic reviews and clinical trials, supporting the generalizability of these results [3,4].

Repeated-measures analysis demonstrated statistically significant differences across time points for all sites (p < 0.001), with large effect sizes (partial η² = 0.78-0.89). WC showed a moderate positive correlation with reductions in subcutaneous fat thickness (r = 0.62-0.71, p < 0.01).

The present study (N = 50) was not powered to precisely estimate or detect rare adverse events. Complication data are therefore reported descriptively and qualitatively compared with published rates. This limitation is typical of single-center observational surgical series and should be acknowledged.

## Discussion

The present study demonstrates that large-volume abdominal liposuction, assessed using USG, significantly reduces subcutaneous fat thickness and WC at six months post-procedure, with high patient satisfaction and minimal complications. These findings are consistent with recent literature supporting the reliability and validity of USG for quantifying changes in subcutaneous fat after body-contouring interventions. In this study, patients were aged 21-45 years and underwent large-volume abdominal liposuction (>5 L of aspirate). Some previous studies did not clearly specify the type or volume of liposuction performed. Additionally, the present study evaluated the time course of postoperative swelling and changes in abdominal fat characteristics following large-volume liposuction. Patients undergoing liposuction in other body areas or concomitant surgical procedures were excluded to maintain homogeneity. Unlike earlier studies that did not measure subcutaneous fat thickness at multiple abdominal sites, our study assessed thickness at four predefined points (S, I, R, L) preoperatively and at one week, one month, and six months postoperatively.

A 2018 systematic review provided a comprehensive overview of fat quantification techniques, including USG, and emphasized the importance of controlling probe pressure and using consistent anatomical landmarks to ensure reproducibility [5]. A 2025 scoping review by Neagu and Neagu further highlighted USG as a reliable and valid method for measuring subcutaneous fat thickness at selected anatomical sites [6]. USG also allows detection of intra-abdominal pathology, such as hernias, cysts, or tumors, and can serve as an objective tool for surgical planning and postoperative assessment [7]. A 2024 study supported its technical validity for body fat quantification, demonstrating strong correlation with computed tomography (CT) in selected clinical populations [8]. Hoffmann et al. reported that USG offers advantages over skinfold calipers and shows reliability comparable to magnetic resonance imaging (MRI) for assessing abdominal subcutaneous fat [7]. Although CT and MRI remain the gold standards for precise fat-volume quantification [9], their higher cost, limited accessibility, and radiation exposure (in the case of CT) restrict their routine postoperative use.

Nearly all patients demonstrated fluid collections on ultrasonography at one week postoperatively, most prominently in the flank region. Most edema resolved by one month, and no persistent collections were observed thereafter. Significant reductions in subcutaneous fat thickness at all four sites (S, I, R, L) were maintained at six months. The early maximum reduction followed by partial rebound at one month reflects expected edema resolution, while stabilization at six months confirms durable fat removal and tissue remodeling. The greatest reduction at the L site (44.14%) may reflect individual anatomical variation in fat distribution. This site-specific variation highlights the importance of individualized surgical planning.

WC was measured only at the six-month follow-up, demonstrating an average reduction of 9.82 cm, approximately 10% of the preoperative value. This represents a clinically meaningful decrease in central adiposity and supports the contouring efficacy of liposuction. Early postoperative circumferential measurements were intentionally avoided because edema, tissue inflammation, seroma formation, and compression garment use can significantly influence measurements. By six months, tissue stabilization allows WC to serve as a reliable indicator of the final contour outcome. The weak and non-significant correlations between reductions in WC and ultrasonographic fat thickness emphasize an important methodological distinction. WC is a global anthropometric parameter influenced by visceral fat, abdominal wall tone, posture, and fluid shifts. In contrast, ultrasonography provides a direct, localized measurement of subcutaneous fat at predefined anatomical sites. The absence of strong correlation therefore supports the superior precision of ultrasonography for detecting site-specific changes, particularly during early and intermediate recovery.

The heaviest patient enrolled weighed 96 kg with a WC of 116 cm. At six months, her weight decreased to 90 kg and WC to 96 cm. Subcutaneous fat thickness decreased by more than 40% at all measured sites (S: 49.5 to 28 mm; I: 59 to 25.4 mm; R: 56.6 to 30.2 mm; L: 66.4 to 31.9 mm). These findings illustrate the magnitude of fat reduction achievable with large-volume liposuction. Sex-based differences in fat distribution were also observed: males tended to have greater supra-umbilical thickness, whereas females demonstrated greater infra-umbilical thickness, while flank distribution was comparable. 

Postoperative bruising was minimal and primarily observed in the flanks and suprapubic region, resolving within 2-3 weeks. Postoperative skin induration softened by the fourth week. Fluid leakage from stab incision sites was greatest on POD 1 and decreased to minimal levels within 2-3 days. No major complications were observed, and all seromas/hematomas resolved by one month. This safety profile aligns with published large-volume liposuction series and meta-analyses [4,10-12], which consistently report low overall complication rates. No cases of skin necrosis or contour deformity occurred in this cohort, further supporting procedural safety in appropriately selected patients with truncal obesity. 

Several potential confounding factors should be acknowledged, including postoperative diet, exercise, daily activity levels, lifestyle factors, body mass index, socioeconomic status, and compliance with pressure garment use. In this study, all patients were advised to wear compression garments beginning two days postoperatively and to resume routine daily activities the following day. Working patients were instructed to return to work within 2-3 days. Despite these variables, 96% of patients reported satisfaction with the procedure. Many patients also reported improved self-esteem and increased physical activity following surgery.

Limitations

This study has several limitations. The sample size of 50 patients may restrict generalizability, as the cohort may not fully represent broader variations in age, sex, ethnicity, or body habitus. The absence of a control or comparator imaging modality (such as CT or MRI) limits the ability to validate ultrasonographic measurements against more objective volumetric assessments. Although USG is non‑invasive and widely accessible, it remains operator‑dependent, and factors such as probe pressure, angle, and user experience may introduce measurement variability despite standardization efforts. The six-month follow‑up period, while adequate for early postoperative assessment, does not allow evaluation of long‑term fat redistribution, durability of contour changes, or metabolic effects. Additionally, the study focused exclusively on subcutaneous fat thickness and did not assess visceral fat or overall body composition [13].

## Conclusions

Large‑volume abdominal liposuction produced a consistent and measurable reduction in subcutaneous fat thickness and WC at six months in all patients. USG proved to be a reliable, accessible, and economical tool for objectively quantifying these changes, demonstrating approximately 40% reduction in abdominal fat thickness and about 10% reduction in WC at six months, with postoperative fluid collections resolving by one month and no major complications observed. The weak correlations between WC reduction and ultrasonographic measurements further emphasize that circumferential measures alone may not accurately reflect localized fat‑thickness changes. While CT and MRI remain the gold standards for volumetric assessment, their limited accessibility and higher cost restrict routine use. USG, therefore, represents a valuable, accessible, and clinically meaningful modality for assessing liposuction outcomes, particularly in settings where repeated, site‑specific assessment is required. Future studies with larger cohorts, longer follow‑up, and comparative imaging will help further validate its role in postoperative body‑contouring assessment.
